# A cluster randomized trial to assess the impact of clinical pathways for patients with stroke: rationale and design of the Clinical Pathways for Effective and Appropriate Care Study [NCT00673491]

**DOI:** 10.1186/1472-6963-8-223

**Published:** 2008-11-03

**Authors:** Massimiliano Panella, Sara Marchisio, Antonella Barbieri, Francesco Di Stanislao

**Affiliations:** 1 Department of Clinical and Experimental Medicine, University of Eastern Piedmont "A. Avogadro", Novara, Italy; 2 Department of Hygiene and Public Health, University "Politecnica delle Marche", Ancona, Italy

## Abstract

**Background:**

Patients with stroke should have access to a continuum of care from organized stroke units in the acute phase, to appropriate rehabilitation and secondary prevention measures. Moreover to improve the outcomes for acute stroke patients from an organizational perspective, the use of multidisciplinary teams and the delivery of continuous stroke education both to the professionals and to the public, and the implementation of evidence-based stroke care are recommended. Clinical pathways are complex interventions that can be used for this purpose. However in stroke care the use of clinical pathways remains questionable because little prospective controlled data has demonstrated their effectiveness. The purpose of this study is to determine whether clinical pathways could improve the quality of the care provided to the patients affected by stroke in hospital and through the continuum of the care.

**Methods:**

Two-arm, cluster-randomized trial with hospitals and rehabilitation long-term care facilities as randomization units. 14 units will be randomized either to arm 1 (clinical pathway) or to arm 2 (no intervention, usual care). The sample will include 238 in each group, this gives a power of 80%, at 5% significance level. The primary outcome measure is 30-days mortality. The impact of the clinical pathways along the continuum of care will also be analyzed by comparing the length of hospital stay, the hospital re-admissions rates, the institutionalization rates after hospital discharge, the patients' dependency levels, and complication rates. The quality of the care provided to the patients will be assessed by monitoring the use of diagnostic and therapeutic procedures during hospital stay and rehabilitation, and by the use of key quality indicators at discharge. The implementation of organized care will be also evaluated.

**Conclusion:**

The management of patients affected by stroke involves the expertise of several professionals, which can result in poor coordination or inefficiencies in patient treatment, and clinical pathways can significantly improve the outcomes of these patients. It is proposed that this study will test a new hypothesis and provide evidence of how clinical pathways can work.

**Trial Registration:**

ClinicalTrials.gov ID [NCT00673491]

## Background

Stroke represents one of the major public health problems worldwide. Despite advances in stroke prevention, diagnosis, treatment, and rehabilitation, stroke causes 9% of all the deaths around the world and stroke-related disability has been judged to be one of the most common causes of disability. In industrialized countries stroke remains a major cause of acute hospitalization with mortality rates at 28 days following the acute onset at approximately 20%. This accounts for more than 4% of direct health-care costs [1-3].

The American Stroke Association's Task Force on the Development of Stroke Systems outlined that the obstacles in translating scientific advances into clinical practice for stroke are often related to a fragmentation of stroke-related care caused by inadequate integration of facilities and professionals that should closely collaborate in providing stroke care. This potentially contributes to the high morbidity, mortality and economic cost of this disorder [4]. Other studies also suggest that establishing well-organized and multidisciplinary stroke care can help improve the quality of service delivered and reduce stroke mortality rates [5,6].

According to the Helsingborg Declaration in 2006 on European Stroke Strategies, all patients in Europe with stroke should have access to a continuum of care, from organized stroke units in the acute phase through to appropriate rehabilitation and secondary prevention measures. Consequently to improve the outcome for acute stroke patients, the optimization of the use of multidisciplinary teams, the development of better ways to deliver a continuing stroke education to the professionals and to the public, the implementation of evidence-based stroke care and the evaluation of different models of stroke services were identified as research and development priorities in stroke care from an organizational perspective [7].

A recent review showed that clinical pathways are tools that can help to achieve these goals when applied to stroke care [8]. Clinical pathways are complex interventions for the mutual decision making and organization of care processes for a well-defined group of patients during a well-defined period with the aim of enhancing the quality of care across the continuum by improving risk-adjusted patient outcomes, promoting patient safety, increasing patient satisfaction, and optimizing the use of resources. Their defining characteristics also include an explicit statement of the goals and key elements of care based on evidence, best practice, and patient expectations; the facilitation of communication amongst the team members and with patients and families: the coordination of the care processes by coordinating the roles, and sequencing the activities of the multidisciplinary care team, patients and their relatives; the documentation, monitoring, and evaluation of variances and outcomes; and the identification of the appropriate resources [9].

The purpose of the Clinical Pathways for Effective and Appropriate Care Study was to determine whether clinical pathways could improve the quality of the care provided to patients who have been affected by stroke both in hospital and through the continuum of the care.

### Objectives

The main objective of the trial is to evaluate the effectiveness of the implementation of clinical pathways for acute stroke care and rehabilitation within a sample of Italian hospitals and rehabilitation long-term care facilities. The hypothesis is that clinical pathways would be more effective than usual care in treating stroke patients and that the clinical pathways would reduce both patient mortality and improve patient outcomes.

The secondary objective is to determine whether clinical pathways increase the appropriateness of the care provided to the patients in hospital and through the continuum of the care and its effects on the outcomes.

The third objective is to determine whether clinical pathways help to implement organized care in acute stroke care and rehabilitation facilities and its effects on the outcomes [5].

A pilot study to define baseline levels of performance in acute stroke care and rehabilitation facilities has been described previously in a separate protocol [10].

## Methods

### The Project

The Clinical Pathways for Effective and Appropriate Care Study was promoted and funded by the Italian Ministry of Health (Special Programs art. 12 bis D.lgs 229/99) and Marche Region. The funding sources played no role in the design, conduct, analysis, interpretation, or reporting of the study. The study's Steering Committee defined the study's objectives, clinical topics, scheduling and design. The Regional Healthcare Agency of Marche Region coordinated the project and provided administrative support.

### Study design

The Clinical Pathways for Effective and Appropriate Care Study was designed as a multi-centre cluster randomized controlled trial, in which the experimental group contains stroke patients treated according to specific clinical pathways, while the control group received usual care. A cluster design was used (with single hospitals and rehabilitation long-term care facilities of the same area as the randomization units) because of the Ethical and logistical issues associated with the implementation of clinical pathways, which involves a series of complex actions at institutional level [11-14].

### Randomization

**Figure 1:**
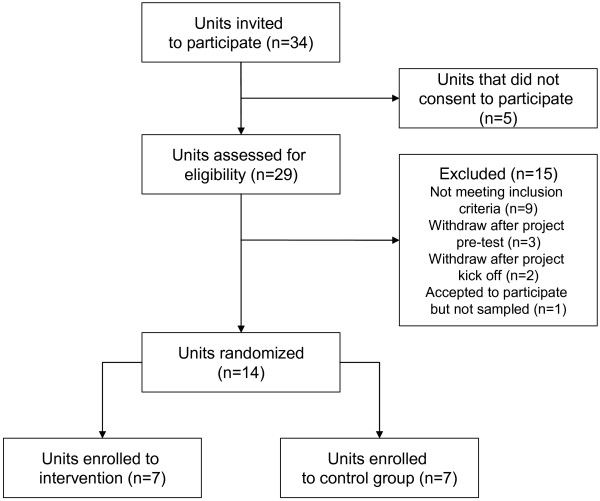
Flow diagram of the progress of the units through the trial.

Thirty four units based in nine Italian Regions were invited to participate in the study (figure 1). Twenty nine units (hospitals and rehabilitation long-term care facilities within the same area) showed interest in the implementation of the clinical pathways for the acute care and rehabilitation of stroke and were assessed for eligibility. The selection of the units was based on the comparability of their location, patient population, facilities and teaching status both for hospitals and rehabilitation long-term care facilities. To participate in the study the administrators of the hospitals and the rehabilitation long-term care facilities had to allow the institution to be allocated to either of the two strategies (clinical pathway or current practice) for a 1-year period and agree not to implement a clinical pathway for the acute care and/or rehabilitation of stroke if assigned to the usual care group.

Nine units were excluded because they did not meet the inclusion criteria and five units withdrew after the project commenced following a decision by their management (three units withdrew after project pre-test and two units withdrew after project kick off). One unit could not provide reassurance that they would not implement a pathway if assigned to the control group and therefore they were not included in the study sample. The remaining units were randomized to either of the two arms. A simple randomization was carried out before the intervention and patient recruitment started, using a computer-generated sequence with allocation concealment. Fourteen units were selected and randomized. Blinding of patients and clinicians was not possible.

### Study evaluations

The primary outcome measure was patient mortality 30-days following the stroke. Thirty-day mortality was defined as the proportion of ischemic stroke events that are fatal within 30 days from the onset. Stroke fatality was chosen as main outcome because it is clinically relevant, objectively measured, and reliably coded [5]. The impact of the clinical pathways, comparing the length of the in-hospital stay, the hospital re-admissions rates, the institutionalization rates after hospital discharge, the patients' dependency levels, and complication rates along all the continuum of the care were also analyzed. The list of outcome indicators is reported in table 1.

**Table 1:** The outcome indicators set

**Indicator**	**Measure**	**Criterion met/expected change**	**Source, Year**
In-hospital death rate within 30 days from admission to hospital	(%)	Differences in rates	Clinical Outcome Working Group, 1995, 1997; NHS Centre for Coding and Classification, 1990
In-hospital death rate within 30 days from stroke attack	(%)	Differences in rates	
Post-discharge death rates (1, 3, 6, 12 months after discharge)	(%)	Differences in rates	
In-patients length of stay	Days	Differences in means	Schmidt WP, 2003
Within 9 days length of stay in hospital patients' rate	(%)	Differences in rates	
Pressure ulcers incidence rate	(%)	0%	Clark M, 1991; Effective Health Care, 1995
Overall in-hospital complications rate	(%)	4%	Adams HP Jr, 2003
Overall post-discharge complications rate	(%)	Differences in rates	
Dependency at discharge	FIM scale	Differences in means	Gompertz P, 1993 Wade DT, 1987
Dependency at 6 months after stroke	FIM scale	Differences in means	Hardwood RH, 1994
Institutionalization at discharge	(%)	Differences in rates	Nikolaus T, 2000 Kalra L, 1995
In-hospital re-admission rate (within 30 days from discharge)	(%)	Differences in rates	Ebrahim S, 1987 Milne R, 1990
Return to pre-stroke functioning in daily life rate (with ADL/case mix adjustment)	(%)	Differences in rates	Early Supported Discharge Trialists (Cochrane 2005, Issue 2)

The quality of the care provided to the patients was assessed by monitoring the use of diagnostic and therapeutic procedures during hospital stay and rehabilitation, and by the use of key quality indicators at discharge as reported in previous studies (table 2). The implementation of organized care was also evaluated (table 3).

**Table 2:** The process indicators set

**Indicator**	**Measure**	**Criterion met/expected change**	**Source, Year**
Information, advice and support from the multidisciplinary team given to the patients (and with their consent, to the carers)	(%)	Given to all the patients/relatives/care givers	SIGN 64, 2002
Use of referral protocols (to neurovascular clinics and admission to stroke unit)	(%)	Given to all the patients	NHS QIS (CSBS-PPI 2002) Clinical Standards, 2004; CHD/Stroke Task Force, 2001; SPREAD, 2005
Use of clinical protocols (at least 5 of the following)	(%)	Given to all the patients	CHD/Stroke Task Force, 2001; CSBS 2002; Antiplatelet Trialists' Collaboration, 1994; SIGN 13, 1997; European Atrial Fibrillation Trial, 1993; Hebert PR, 1997; SIGN 14, 1997; Antithrombotic Trialists' Collaboration, 1998
- Stroke treatment/management protocols			
- Antiplatelet/anticoagulant protocol			
- Diabetes treatment protocol			
- Atrial fibrillation therapy protocol			
- Blood pressure lowering protocol			
- Cholesterol lowering protocol			
- Suspected carotid stenosis protocol			
- Smoking cessation protocol			
Use of (local) admission to social services protocols	(%)	Given to all patients	Report to the Dept. of Health, 2000
Use of CT/MRI brain scan within 48 hours from admission	(%)	80%	SIGN 14, 1997; Wardlaw JM, 2003
Aspirin treatment within 48 hours from admission	(%)	Given to all patients	Gubitz G (Cochrane 2003)
Swallow screen test on day of admission	(%)	Given to all patients	SIGN 20, 1997
Blood pressure assessment	(%)	Given to all patients	Progress, 2001; NHS QIS (CSBS 2002); Clinical Standards, 2004
ECG/ECD within 24 hours from admission	(%)	Given to all patients	SIGN 13, 1997
Continuous monitoring within 48 hours from admission (see parameters below)	(%)	Differences in rates	SIGN 13, 1997; New Zealand Guidelines Group, 2003; Canadian Stroke Network and the Heart and Stroke Foundation of Canada, 2006
- Blood pressure			
- Glycaemia			
- Electrolitemia			
- Breath			
Before discharge total assessment (see parameters below)	(%)	Given to all patients	MRC/BHF Heart Protection Study, 2002, 2003; SIGN 24, 1998
- Tobacco smoke			
- Lipemia			
- Glycaemia			
Use of discharge plan (and communication)	(%)	Given to all the patients/relatives/care givers	SIGN 24, 1998
Use of SIGN guidelines-based discharge plan	(%)		SIGN 24, 1998
Use of discharge summary and information (information pack)	(%)		RCPE, Consensus Panel, Nov 2000; SIGN 65, 2003

**Table 3:** The organized care indicators set

**Indicator**	**Measure**	**Criterion met/expected change**	**Source, Year**
Use of organized care	OCI	Differences in rates	Saposnik G, 2007
Admission to stroke unit	(%)	100%	NHS QIS (CSBS 2002) Clinical Standards, 2004; CHD/Stroke Task Force Report, 2001; SIGN 13, 1997; SIGN 64, 2002
Stay in stroke unit within 24 hours after admission and until the end of in-hospital rehabilitation	(%)	70%	
Use of case managers (physiotherapists, occupational therapists, nurses specialized in stroke care)	(%)	Given to all the patients	RCP IWP/S, 2000; SIGN 64, 2002
Use of stroke team	(%)	Given to all the patients	CHD/Stroke Task Force, 2001; SIGN 64, 2002
Assessment for rehabilitation needs by a member of the stroke team within 48 hours after admission	(%)	Given to all the patients	Brown M, 2000
Patients' needs assessment and planning rate for post-discharge services	(%)	Given to all the patients	RCPE, Consensus Panel, Nov 2000; NHS QIS Clinical Standards, 2004
Follow-up rate within 3 months after discharge (by specialist/stroke team)	(%)	Given to all the patients	

### Study subjects

The sample included all patients admitted to the hospitals during the experimental period with a principal diagnosis of acute ischemic stroke (ICD-9CM code 434.91) and a minimum age of eighteen. A baseline was verified by comparing the two groups on the admission measuring patients' age, sex, co-morbidities (based on the Charlson-Deyo index), risk factors (including smoking, diabetes, preexisting heart diseases, hyperlipidemia and hypertension) and symptom severity (using the Functional Independence Measure – FIM scale). Patients with hemorrhagic strokes (all ICD-9CM codes included in 431.xx code) and transient ischemic attacks (ICD-9CM code 435.9) were excluded.

### Study sample

A calculation was made to identify the sample size needed to detect a statistical difference in the 30-days mortality rate. As between 8% to 17% of ischemic stroke patients die within 30 days of the incident, it was expected that the clinical pathways would succeed in limiting mortality to 8% and would therefore be clinically relevant [3,15,16]. Based on this goal a sample size of 476 patients (238 in each group) was required for the study to have 80% power at the 5% significance level. The sample size calculation was performed according to standard criteria for cluster randomized trials. The sample size was adjusted using an inflation factor of 1.43 to account for the cluster randomization: 7 clusters per trial arm, cluster size of 24 patients, ICC of 0.018 [17-19].

### Intervention

The project started at each hospital with a grand round that outlined the project protocol. One physician or nurse with at least two years of experience of clinical pathways was assigned to each hospital in the experimental group, in order to facilitate project implementation (this included staff education in the use of the clinical pathway). The teams included internal medicine physicians, neurologists, physiatrists, epidemiologists, physiotherapists, occupational therapists, nurses, hospital pharmacists, psychologists, social workers and support staff. The teams were formed on a voluntary basis, they received three days training in the development of clinical pathways and then they developed their clinical pathway over a 6-month period. All groups analyzed their care processes, reviewed best evidence (this was provided by senior investigators), defined the appropriate goals of the pathways, compiling the results into protocols and documentation. This included the sequencing of events and expected progress of the patients over time [9,20].

The clinical pathways were analyzed by the EBM unit of the Regional Healthcare Agency of Marche and they were judged consistent with current recommendations for the diagnosis and the treatment of stroke. After the validation of the pathways each unit team educated its staff in the use of the clinical pathway and monitored the use of the pathway. This meant that the clinical pathways used in the study were not completely identical because of organizational adaptations at some sites. However, they all substantially adhered to the existing Italian guidelines on the hospital treatment for stroke [21].

### Data analysis

Data was prospectively collected by local staff for both the interventions and the control groups (physicians and nurses had been trained prior to commencement of the study at two educational events in order to do this). Incentives for the local staff were not used. Data was collected using a standardized data extraction instrument which utilized web technology. This allowed only data without unique personal identifiers, but with a unique study identification code (and therefore anonymous), to be entered into a secure database housed at the University of Eastern Piedmont.

The analysis will be performed by the research team. In addition to common descriptive statistics (Fisher exact and Kruskal Wallis test for categorical and continuous variables, respectively), that will be performed at the cluster level, the differences in the rate of 30-days mortality across groups and according to each variable under study will be evaluated using random-effects logistic regression, thus accounting for the clustering effect [5,22,23]. Variables will be included if significant at the 0.10 level (backward approach), with the exception of age which will be forced to entry. The presence of multicollinearity, interaction and higher power terms will be assessed to check final model validity.

Statistical significance will be defined as a two-sided p-value < 0.05. All analyses will be intention-to-treat and will be carried out using Statistica statistical software.

### Ethics

The project received ethical clearance as a prerequisite of approval for funding from the Italian Ministry of Health, according to the Italian Ministry of Health law number (ex art. 12bis D.lgs 229/99). The managers in each unit have consented to their clinic taking part in the trial. Patient's consent to be randomized to the intervention or control arms has not been obtained, because according to the study design randomized occurred at the unit level. However all individual patients gave consent to participate in the study and had the opportunity to withdraw from the study at any time. All patients' data was managed according to the Italian Data Protection act.

## Discussion

The aim of this study is to improve the quality of care through clinical pathways and thus should not imply any risk for the patients affected by the study. It is difficult to imagine that interventions based on better evidence and appropriate use of technologies and drugs could worsen the quality of care when compared to usual care. However despite continuing international interest in implementing clinical pathways, the evidence base for their effectiveness in improving the quality of stroke care is still inconclusive [8].

Clinical pathways could be defined as a complex intervention in which a number of separate elements are essential to the functioning of the intervention, but the "active ingredient" that is effective is difficult to specify [11]. For this reason it could be problematic to evaluate properly clinical pathways mainly because of the difficulty of keeping the intervention replicable and recognizable. However we think that a cluster randomized controlled trial design is the most appropriate to evaluate clinical pathways [24]. To standardize the intervention we defined as standard the steps in the change process or the key functions that the elements of the intervention were meant to improve according to each context. Moreover our indicators were driven by the theory and concerned the functions provided by the key elements of the clinical pathways that were based on expected adherence to the same evidences. So we think that this helped to keep the integrity of the intervention in each site [10,25].

We also think that the outcome measures chosen were well-suited to measuring the impact clinical pathways have on stroke care, because they were measures that evaluated most of the perspectives of stroke care from acute to rehabilitation settings. In particular two outcome measures – mortality and organized care – can help to investigate the new hypothesis which is to understand how clinical pathways can work. In fact the management of patients affected by stroke involves the expertise of several professionals, which can result in poor coordination or inefficiencies in patient treatment, and clinical pathways can significantly improve the outcomes of these patients. However, the active ingredient of clinical pathways is unclear, so we think that our study will help to better understand which mechanisms within the clinical pathways can really improve the quality of care.

## List of abbreviations

ICD-9CM: International Classification Diseases 9^th ^revision Clinical Modification; ICC: Intra Cluster Correlation; EBM: Evidence Based Medicine; FIM: Functional Independence Measure; ADL: Activity Daily Life; OCI: Organized Care Index.

## Competing interests

The authors declare that they have no competing interests.

## Authors' contributions

MP conceived and developed this study and wrote the manuscript. SM helped to design the study and the manuscript. AB assisted in the cluster creation, in defining the indicator set and contributed to the manuscript. FDS gave input into the project, overviewed all of the steps of the study design and undertook the final review of the manuscript. All authors read and approved the final manuscript.

## Pre-publication history

The pre-publication history for this paper can be accessed here:


